# Palliative TURP Combined with Intermittent ADT Is A Curative Therapy to Some Elderly Men with Localized Prostate Adenocarcinoma

**DOI:** 10.7150/jca.83825

**Published:** 2023-05-07

**Authors:** Xu Zhang, Qier Xia, Jie Xu

**Affiliations:** Deparetment of Urology, Shanghai Pudong New Area People's Hospital, Shanghai, China.

**Keywords:** elderly,male, prostate cancer, transurethral resection of prostate, androgen deprivation therapy, palliative therapy

## Abstract

**Background:** Radical prostatectomy is the preferred therapeutic option for patients with localized prostate adenocarcinoma whose life expectancy is greater than 10 years. But for elderly patients, this may not be the best option. In clinical work, we have observed that palliative transurethral resection of prostate (pTURP) combined with intermittent androgen deprivation therapy (ADT) has achieved significant good results in the treatment of elderly patients with localized prostate adenocarcinoma.

**Methods:** Retrospective analysis was conducted on 30 elderly patients aged 71 to 88 years who were hospitalized for urinary retention from March 2009 to March 2015. These patients were diagnosed as localized prostate adenocarcinoma with stage T1 to T2 and benign prostatic hyperplasia (BPH) through MRI and prostate biopsy. Fifteen cases (group A) were given pTURP and intermittent ADT after surgery. Fifteen cases (group B) were given sustained ADT. Serum total prostate specific antigen (TPSA), testosterone, alkaline phosphatase (ALP), prostate acid phosphatase (PAP), International Prostate Symptom Score (IPSS), Quality of Life (QOL) score, maximum urinary flow rate (Qmax), average urinary flow rate (Qave), prostate volume and post-void residual urine (PVR) data were followed up for 5 years, and the differences between the two groups were compared.

**Results:** The 5-year cumulative survival rate of group A was 100%. Prostate specific antigen (PSA) progression-free survival was 60.00%. The average duration of intermittent ADT was 23.93 months. Prostate volume reduction was significantly. The dysuria in all patients was significantly improved. Nine patients had TPSA lower than 4 ng/ml and had no local progression and metastasis. At the same time the 5-year cumulative survival rate of group B was 80%. PSA progression-free survival was 26.67%. Six cases of dysuria improved. There was no significant difference in serum TPSA, ALP and PAP between the two groups in five years (P>0.05). Serum testosterone, IPSS score, QOL score, prostate volume, Qmax, Qave, and PVR were significantly different between the two groups in five years (P<0.05).

**Conclusion:** pTURP for elderly patients with localized prostate adenocarcinoma and BPH combined with intermittent ADT is an effective treatment. It is able to solve dysuria. The overall ADT time is short. The risk of progression to castrated resistant prostate cancer is low. Some of them have achieved tumor-free survival.

## Introduction

Radical prostatectomy, as the preferred treatment for localized prostate adenocarcinoma in stages T1 and T2, has been chosen by most doctors and patients. However, the most clinically relevant side effect is long-term urinary incontinence after surgery 1. Some other treatment methods targeting local treatment of the prostate also have their own clusters, such as high-intensity focused ultrasound ablation 2, cryotherapy 3, and proton and heavy ion radiotherapy 4.

Due to the frailty of the elderly, multiple complications, short life expectancy, and the risks and complications of radical prostatectomy, some elderly patients and their families are generally reluctant to receive some relatively large trauma treatment. However, they still hope to receive less traumatic palliative treatment or medication to treat tumors, solve urination problems, and improve their quality of life.

Some studies have shown that as a palliative therapy, pTURP has the advantages of small trauma and fast recovery in the treatment of advanced adenocarcinoma with bladder outlet obstruction. pTURP, castration and intermittent androgen block, which can extend the survival time and improve the quality of life of patients 5-7. Can elderly patients with localized prostate adenocarcinoma receive this treatment to achieve better results? We retrospectively analyzed 30 patients with the palliative therapy. It was found that among 15 cases who had undergone pTURP and received intermittent ADT after surgery, 9 cases achieved tumor free survival. Five cases still followed up for more than 8 years. The oldest of whom is 91 years old now. The incidence of urinary incontinence and other complications in all cases receiving pTURP was low and the satisfaction was high. The 5-year follow-up data is now reported as follows.

## Materials and Methods

### Patients and our study

All procedures performed in this study followed the Declaration of Helsinki (as revised in 2013) and were approved by the Ethics Review Board of Shanghai Pudong New Area People's Hospital (No.: SWJW-1602). Clinical information from patients was extracted from the clinical health information system of our hospital.

Inclusion criteria: We identified 30 elderly patients in the period from March 2009 to March 2015. They admitted to our hospital due to urinary retention caused by prostatic hyperplasia. The patient's age at visit was 71 to 88 years old (mean 78.27 years). Their serum TPSA>4ng/ml. All the patients were diagnosed as localized prostate adenocarcinoma with stage T1-T2 and BPH through MRI and prostate biopsy.

Study groups: Fifteen cases (Group A) were underwent pTURP, and were given intermittent ADT after surgery. Fifteen patients (Group B) were surgically castrated (8 cases) or chemically castrated (7 cases), and were given sustained ADT.

Exclusion criteria: Advanced prostate adenocarcinoma with stage T3-T4. Patients who underwent radical prostatectomy, prostate radiation therapy, and hyperthermia during the follow-up period.

We collected the erythrocyte (RBC) and leukocyte (WBC) counts in urine assay in group A at the 3rd, 6th, 9th, and 12th weeks after pTURP to observe the inflammatory response of the prostate wound after surgery and determine whether the wound repair is successful. We collected chest and abdominal CT and pelvic MRI reports to determine whether there was local progression and metastasis. We collected data on prostate volume and PVR from B-ultrasound reports. Data on serum TPSA, testosterone, ALP, PAP were collected for 5 years, as well as IPSS, QOL, Qmax, Qave.

The baseline characteristics of patients before treatment was shown in Table 1.

### pTURP

Prostate tissue was removed using conventional TURP surgical methods by plasma cutting system. If MRI, transrectal ultrasound, and digital rectal examination indicated that the prostate cancer nodules lied in prostatic envelope, the crispy tumor tissue should be removed as much as possible when plasma resection to the affected side, and the capsule tissue can be seen as fibrous tissue or a little fatty tissue. When we resected the affected side, the tumor tissue should be removed as much as possible. Excision of capsular tissue reached fibrous tissue or a little fat tissue.

### ADT

#### Surgery castration

Orchidectomy or excision of testis in tunica albuginea.

#### Chemical castration

Goserelin Sustained-Release Depot for Subcutaneous Injection, 10.8mg (per 12-week) or 3.6mg (per 4-week) subcutaneous injection.

#### Anti-androgen drug

Flutamide tablet 250mg, 3 times/day, take orally.

#### Intermittent ADT

Chemical castration and Anti-androgen drug were performed after pTURP. When serum TPSA less than 0.2ng/ml and lasts for 3 months, Goserelin Sustained-Release Depot for Subcutaneous Injection and Flutamide tablet should be suspended. When serum TPSA is greater than 4 ng/ml, these two drugs are used again.

#### Sustained ADT

The patient was surgically or chemical castrated and treated continuously with anti-androgen drugs.

### Statistical processing

All statistical analyses were performed using SPSS version 26 (SPSS Inc., Chicago, IL, USA). Continuous data are presented as 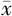
±*s.* Categorical data were presented as numbers and proportions. Fisher's exact test was used to compare the frequency of the two groups. T-test was used to compare the continuous data between the two groups and pre-treatment and post-treatment within the group. The Kaplan-Meier method was used to compare the 5-year cumulative survival curves between the two groups. Cox regression model was used to analyze the progression free survival rate of TPSA in the two groups. Statistical significance was set at *P* < 0.05.

## Results

**Figure 1:**
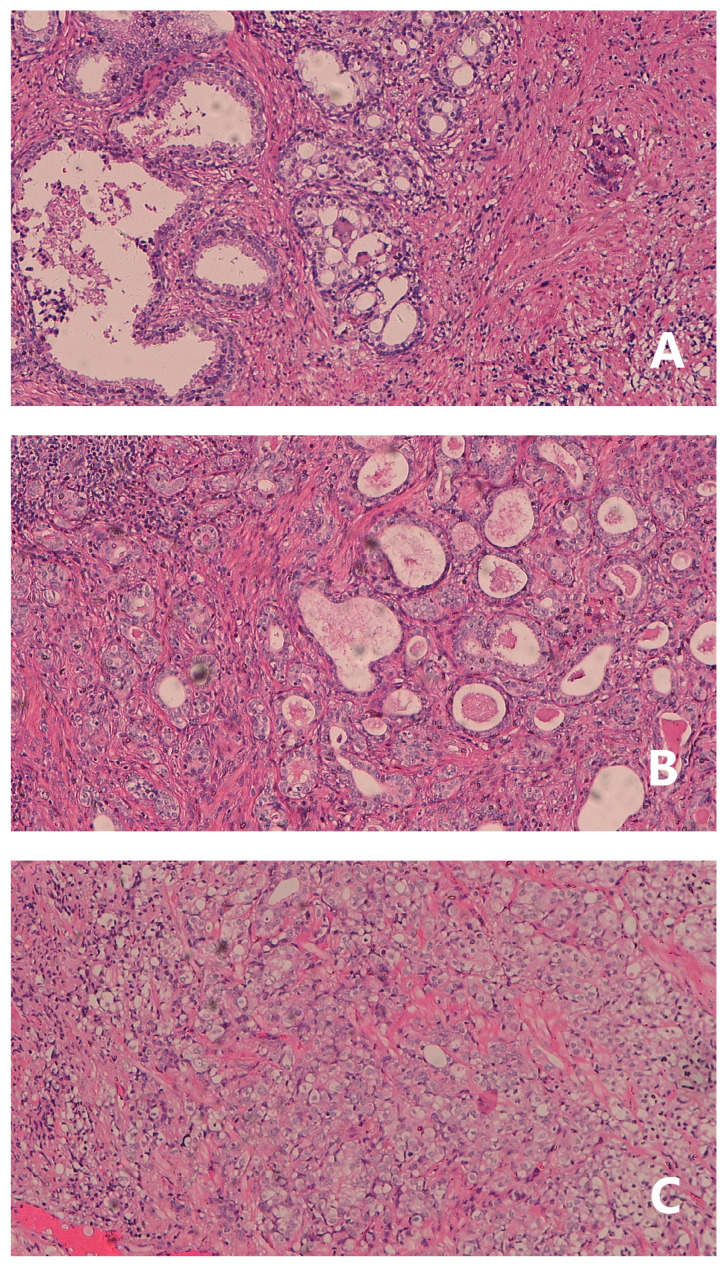
Pathology study of group A of post-pTURP: A prostate adenocarcinoma, Gleason score 3+3=6 point, HE, ×100. B prostate adenocarcinoma, Gleason score 4+3=7 point, HE, ×100. C prostate adenocarcinoma, Gleason score 4+4=8 point, HE, ×100.

**Figure 2:**
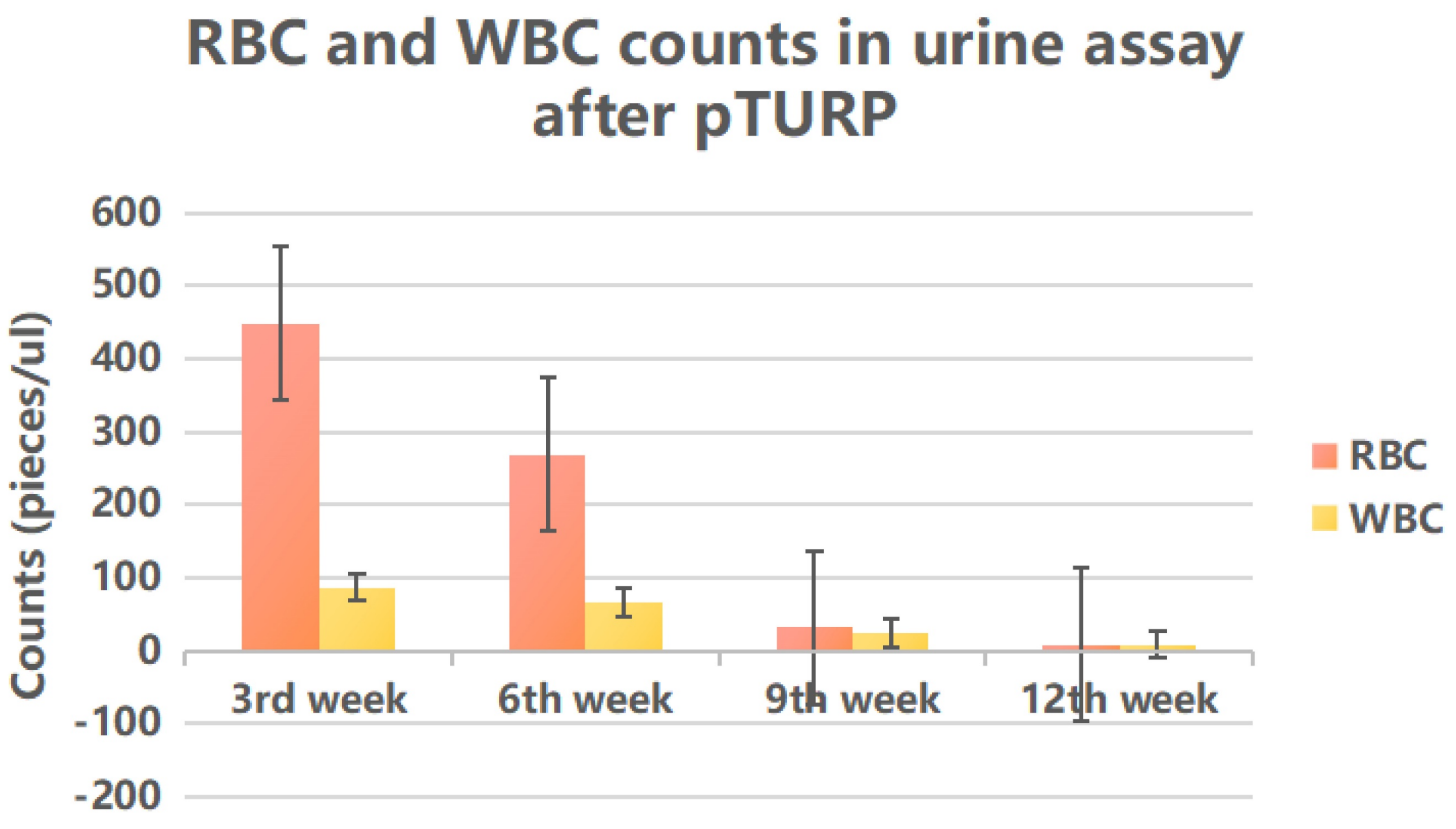
Follow-up of erythrocyte and leukocyte counts in urine assay in group A patients after pTURP.

**Figure 3:**
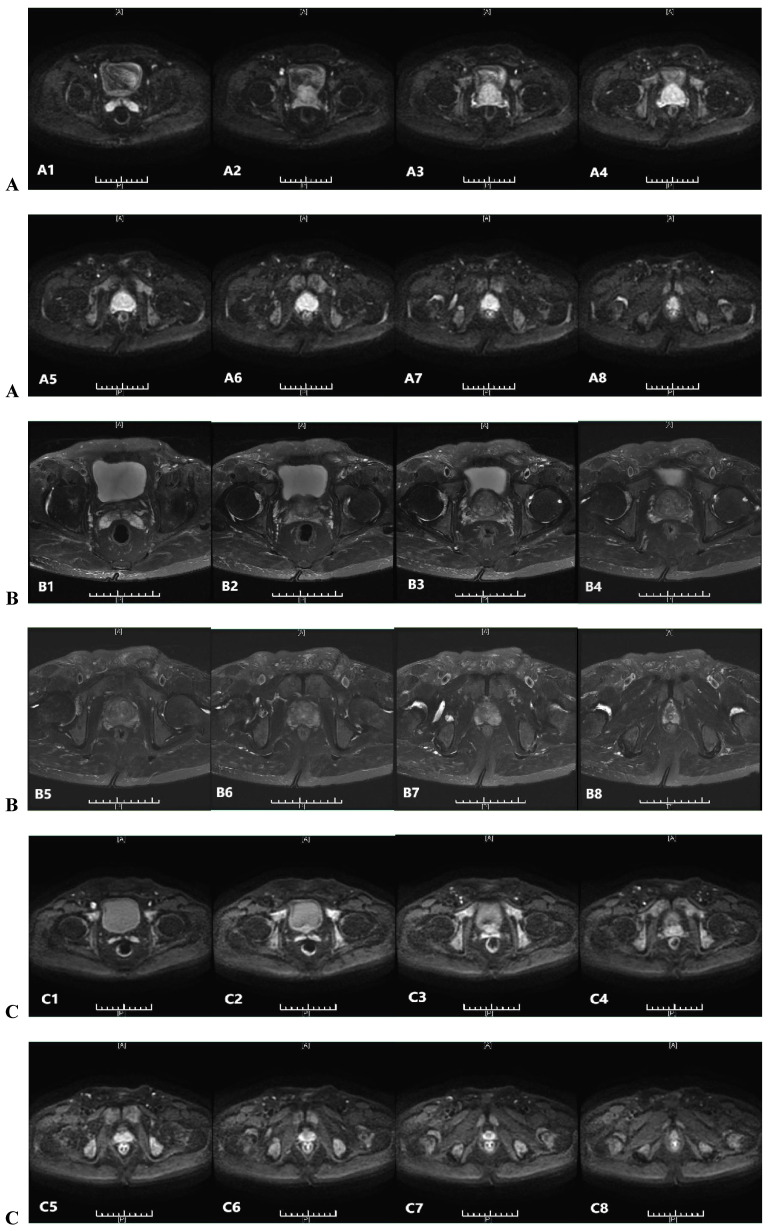

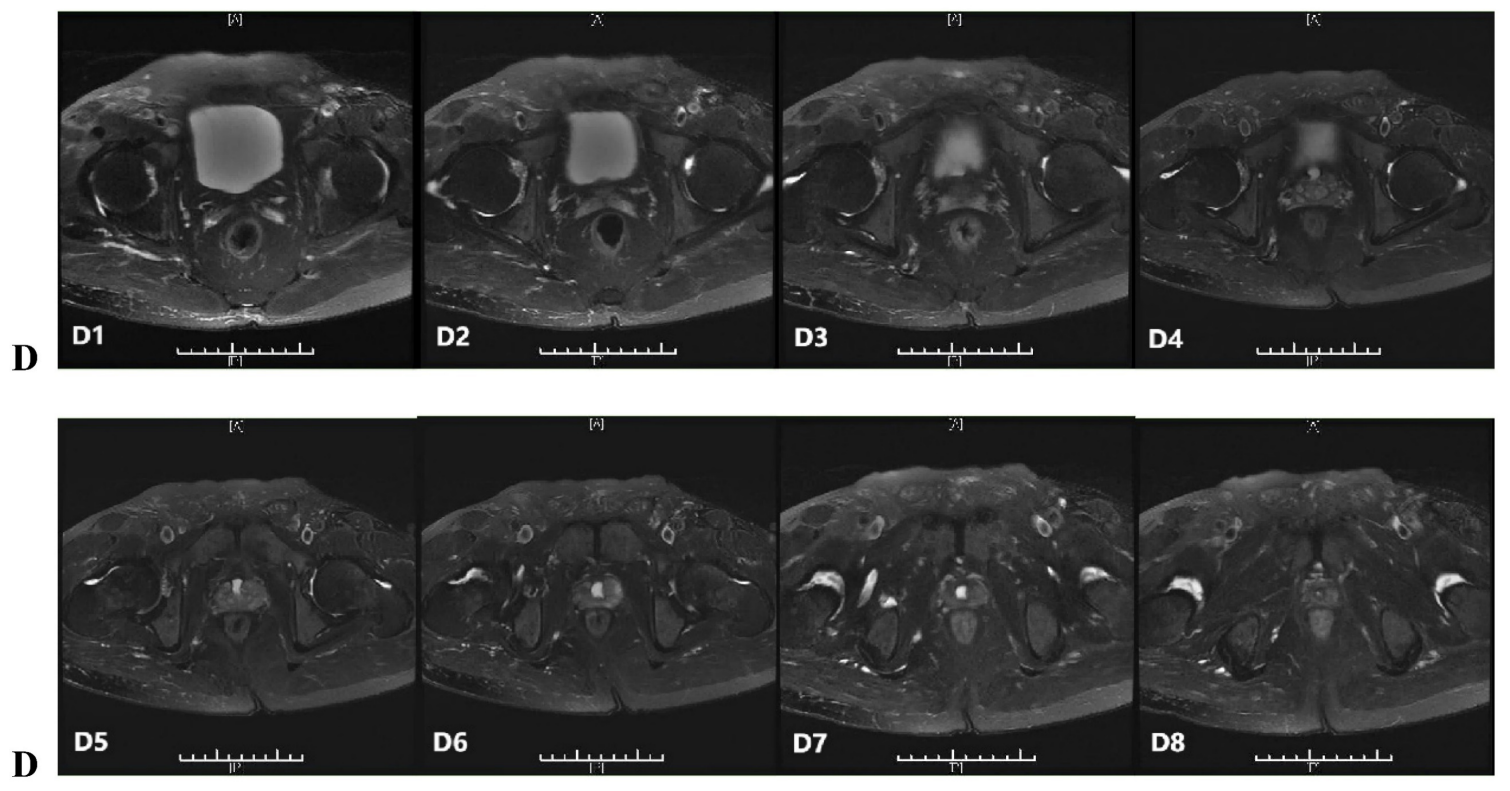
Prostatic MRI of a patient of group A: A(A1-A8) pre-treatment DWI indicate high signal nodule in the prostate. B(B1-B8) pre-treatment T2WI indicate low signal nodule in the prostate. C(C1-C8) post-treatment 5 years DWI indicate no high signal nodule in the prostate. D(D1-D8) post-treatment 5 years T2WI indicate no low signal nodule in the prostate.

**Figure 4:**
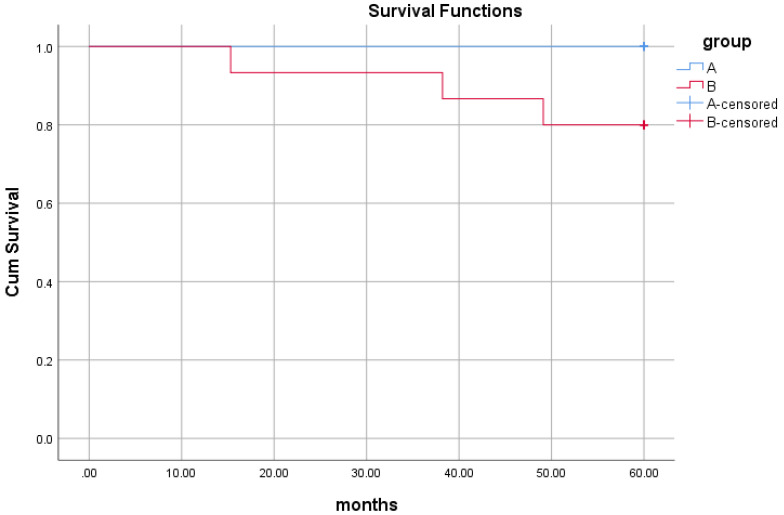
Comparison of 5-year cumulative survival curves of the two groups. Censored Percent of group A was 100%. Censored Percent of group B was 80%. The Log Rank test results of overall comparison of survival curves between the two groups were Chi-Square=3.222, *P*=0.073. Breslow test results were Chi-Square=3.214,* P*=0.073. According to the results of Log Rank test, the survival rate of the two groups of cases was not different.

**Figure 5:**
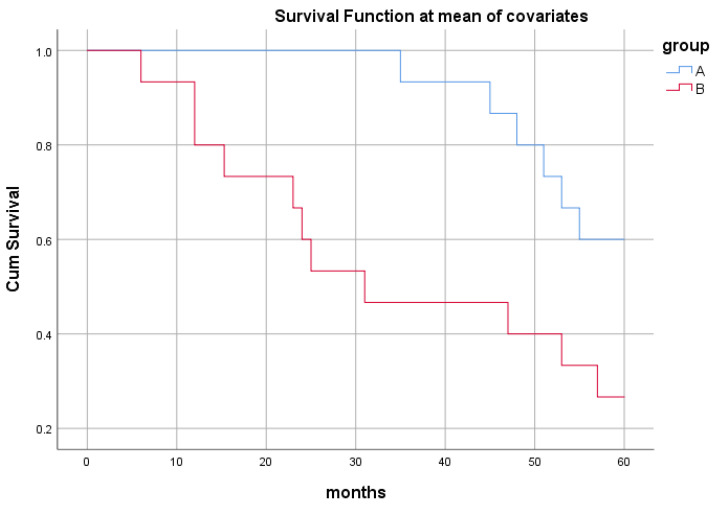
Comparison of 5-year PSA progression-free survival curves of the two groups. Censored Percent of group A was 60.0%. Censored Percent of group B was 26.7%. Cox regression coefficient was b=-4.881,* P*=0.000. The 5-year PSA progression-free survival rate was significant difference between the two groups.

The histopathological study of prostate adenocarcinoma in Group A after pTURP were shown in Figure 1. The comparison of erythrocyte and leukocyte counts at the 3rd, 6th, 9th, and 12th weeks after pTURP in group A patients were shown in Figure 2. Postoperative MRI examination showed that the morphology of the prostate was significantly smaller than that before surgery, as shown in Figure 3. The five-year cumulative survival rate was 100% (15/15), as shown in Figure 4. Nine cases achieved tumor free survival. Their post-operative serum TPSA were lower than 4ng/ml continuously. Their MRI and CT results showed no local recurrence and metastasis. Five cases still followed up for more than 8 years. The oldest of whom is 91 years old now. They didn't use ADT anymore. Five-year PSA progression-free survival was 93.33% (14/15), as shown in Figure 5. The serum TPSA of this PSA progression case was greater than 10ng/ml in the fourth year after surgery. MRI showed that there was a single pelvic metastasis, but there was no fracture. Another case had a little urinary incontinence during daytime activities, but no urinary incontinence at night. One pad/day was needed for him. No complaints of frequent urination and difficulty urinating. According to the re-examination of urodynamics, the patient had detrusor instability and normal bladder capacity. The incidence of urinary incontinence and other complications in all cases receiving pTURP was low and the satisfaction was high. The average intermittent ADT was 23.93 months (95%CI 11.15∼36.72).

In group B, Five-year cumulative survival rate was 80% (12/15). Five-year PSA progression-free survival was 40% (6/15). There were 9 cases of surgery castration. There were 6 cases drug castration. A case died of gastrointestinal bleeding due to thrombocytopenia 12 months after treatment. A case died of lung infection due to chronic bronchitis. A case died of lung infection due to diabetes. Six cases had improved dysuria, three cases required long-term indwelling catheterization, and three cases required intermittent indwelling catheterization. Five cases of post-therapy serum TPSA were lower than 4ng/ml continuously. MRI and CT showed no local recurrence and metastasis. Two cases of serum TPSA was greater than 10ng/ml 1-year later post-therapy. MRI showed that there was a single pelvic metastasis. A case suffered a fracture of the right humerus due to an accidental fall 42 months post-therapy. A case suffered an intertrochanteric fracture of the right femur due to an accidental fall 56 months post-therapy.

**Figure 6:**
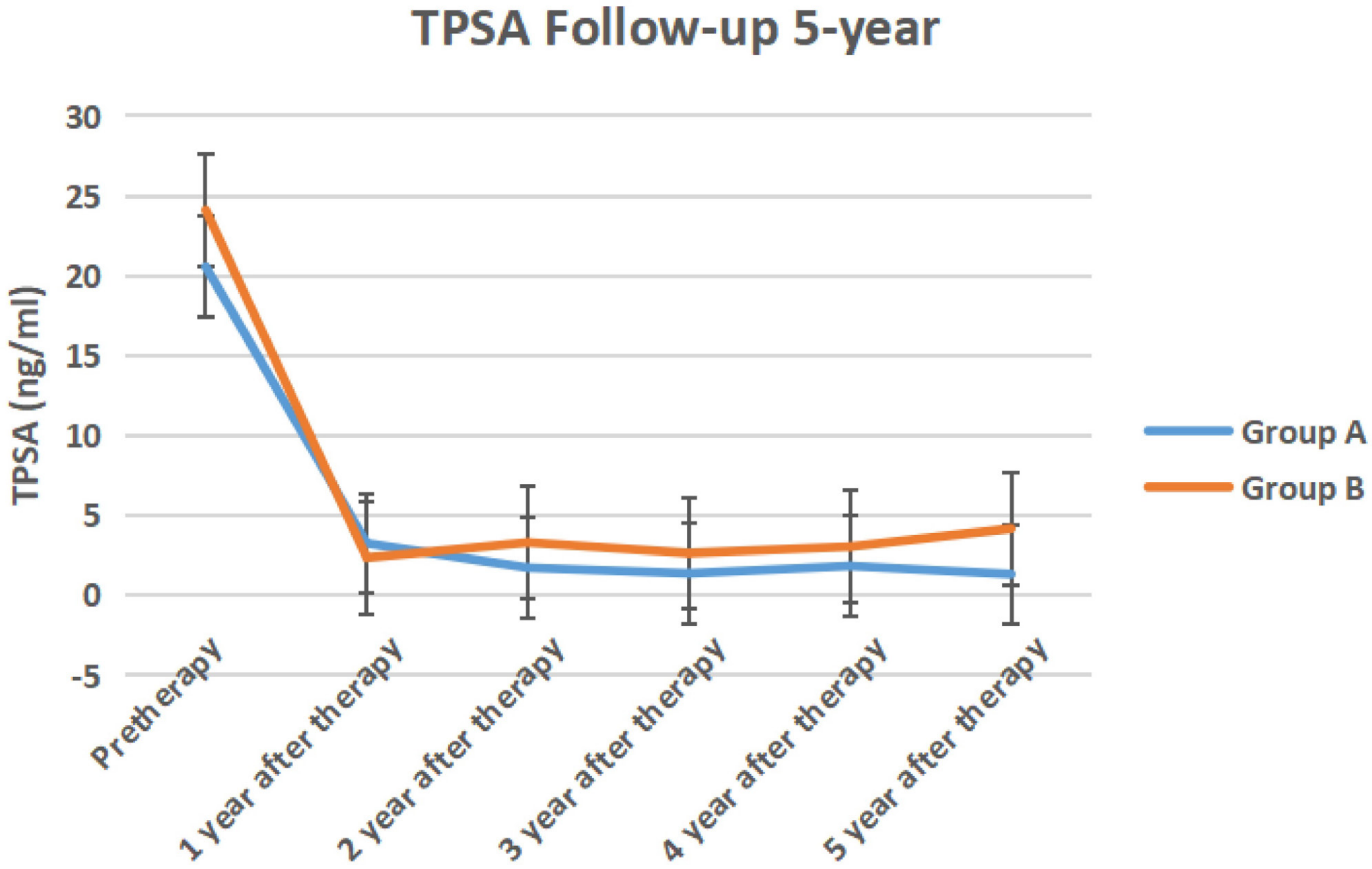
The serum TPSA of the two groups of 5-year follow-up.

**Figure 9:**
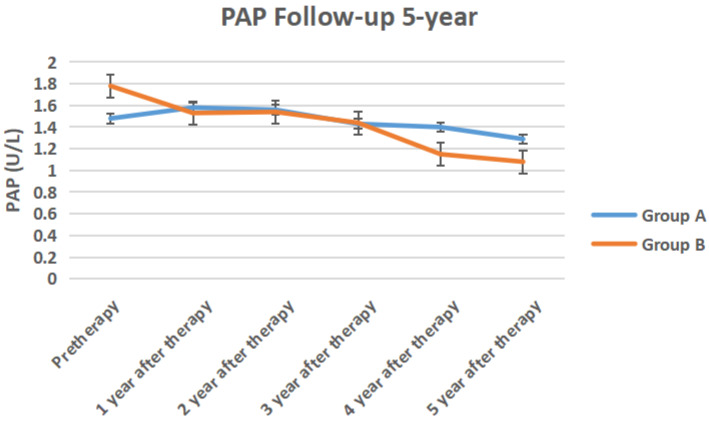
The serum PAP of the two groups of 5-year follow-up.

The serum TPSA, testosterone, ALP, PAP of the two groups of 5-year follow-up were shown in Figure 6 to Figure 9 respectively.

**Figure 10:**
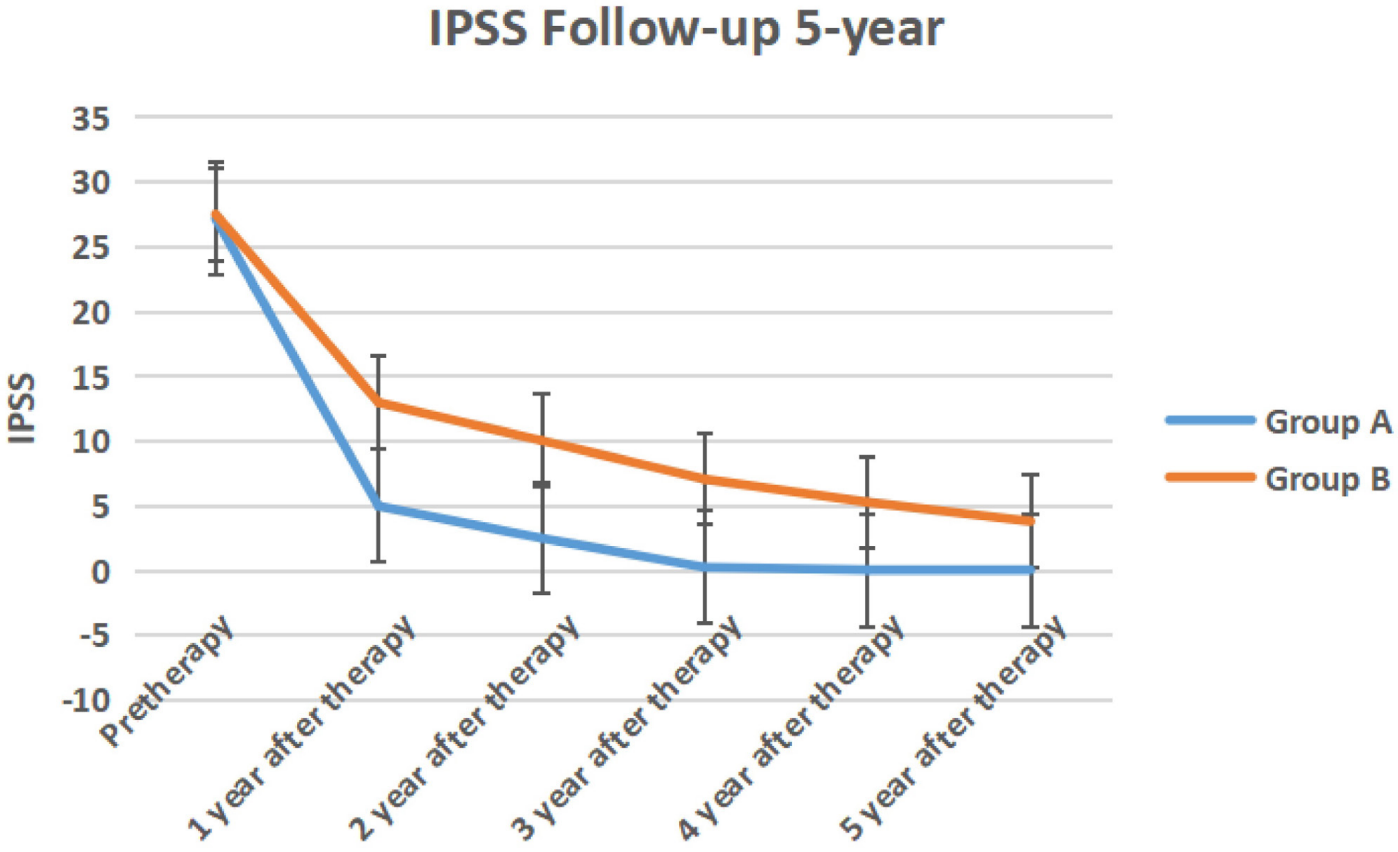
The IPSS score of the two groups 5-year follow-up.

**Figure 15:**
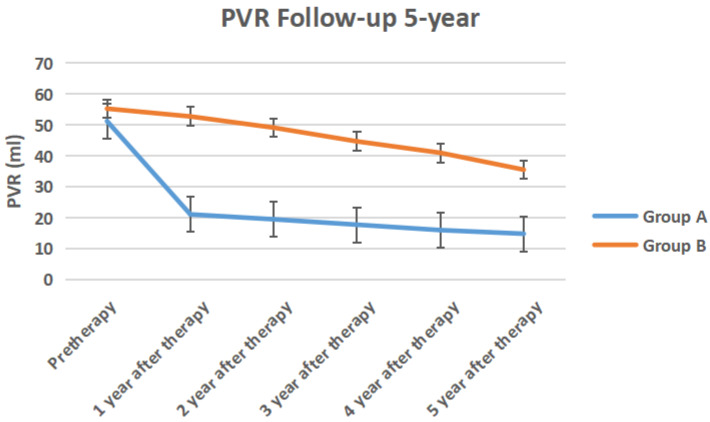
The PVR volume of the two groups of 5-year follow-up.

The IPSS score, QOL score, Qmax, Qave, prostate volume and PVR volume of the two groups of follow-up for 5-year follow-up were shown in Figure 10 to Figure 15 respectively.

## Discussion

With the aging of the urban population, older men are gradually increasing. Many elderly people with prostatic hyperplasia seek medical treatment because of poor urination or even urinary retention. About 20% of these patients have prostate cancer. Prostate cancer morbidity and mortality increase with age and require active response to prolong life 8.

The treatment of prostate adenocarcinoma is mainly for curative purposes, including radical prostatectomy and radiotherapy, with or without androgen castration. Curative treatment options are often accompanied by a high incidence of impotence, urinary tract dysfunction, a significant reduction in quality of life, and high treatment costs, etc. 9-12. Studies have shown that prostate cancer is often not a direct cause of death for the elderly. Patients often die from causes other than prostate cancer. It is believed that the limited prostate cancer with pathological stages of T1 to T2 can choose to wait for observation and follow up closely 13. A meta-analysis shows that radical prostatectomy for prostate cancer can reduce the overall mortality and the risk of prostate cancer-specific death. It should be very cautious in the selection and observation of early localized prostate cancer. The choice of treatment plan needs to be considered in combination with other factors of patients 14.

In our study, a retrospective study was conducted in elderly patients with urinary retention and elevated TPSA with localized prostate adenocarcinoma. Urine assay indicated that the inflammatory reaction in urine continued until 12 weeks after pTURP. The recovery time of pTURP was similar to that of patients with BPH after TURP. There were significant differences between the two groups in prostatic volume reduction, urinary flow rate increase, PVR volume decrease, IPSS and QOL scores descend. None of these cases of group A had bladder clot packing after pTURP, resulting in reoperation to stop bleeding, and no persistent urinary incontinence, urinary retention, nor dysuria. They had smooth urination.

Studies suggest that more than half of castrated resistant prostate cancer patients have detrusor overactivity 15. In our study, only a case had a little urinary incontinence during daytime activities, but no urinary incontinence at night. A pad/day was needed for him. No complaints of frequent urination and difficulty urinating. According to the re-examination of urodynamics, the patient had detrusor instability but normal bladder capacity. He has relieved after intermittent use of the penis clamp, oral M- receptor antagonist treatment.

Therefore, we believe that pTURP was a safe and effective treatment method for prostate adenocarcinoma patients with benign prostatic hyperplasia-induced bladder outlet obstruction.

Because pTURP focuses on further removing the prostate cancer nodules on the basis of TURP, it will inevitably remove part of the capsule tissue containing the tumor, and even reach the fat boundary. The focality of the tumor creates favorable conditions for the surgical resection range. The tumor completely located in the internal gland can be completely removed by the standard TURP because the tumor has not invaded the capsule. However, the tiny tumors are not always loose or crispy like cotton wool when they are removed, and they can be scraped off by pushing with an electric cuting loop, and they are not significantly distinguished from the hyperplastic glands tissue. Tumor lesions can also be seen in the hyperplastic gland tissues and adjacent areas with dense inflammatory cells in the postoperative pathological sections. Furthermore, the tumor located on the capsule, because it has not yet broken through the capsule, the flexibility difference between the fibrous tissue and the edge of the capsule is easy to identify. After the tissue is removed, due to the overall contraction of the prostate capsule when emptying the bladder, small nodules will be significantly raised, which is easy to identify and remove. Because the nodules suggested by the MRI of the prostate before surgery are often different from the tumor sites obtained by puncture, when it is difficult to identify the tumor during surgery, a small part of the capsule in the suspected area can be removed. But it should not be too large to prevent bleeding after vascular injury outside the prostatic fascia. However, the range should not be too large, to difficulty in stop bleeding after vascular injury outside the prostate fascia.

A study of risk-adaptive strategy for the era of active surveillance of prostate adenocarcinoma showed that TURP is used to treat prostate cancer with symptomatic prostate enlargement with a Gleason score of 6. Risk-adaptive transurethral resection of the prostate may prevent overtreatment and allay prostate-specific antigen-associated anxiety in patients with biopsy-proven low-grade prostate cancer and elevated PSA. Additional benefits include voiding symptom improvement and the avoidance of curative therapy's immediate side effects 16. It showed that the status of TURP in the treatment of prostate adenocarcinoma is changing from palliative treatment methods to more active and beneficial treatment measures.

A clinical observation of laparoscopic radical prostatectomy for prostate adenocarcinoma after TURP revealed that postoperative pathology suggested that one patient had no cancer 17. Another clinical analysis of Da Vinci robot-assisted laparoscopic radical prostatectomy for prostate cancer after TURP also showed that in one patient, all specimens were collected postoperatively and no tumors were found. The patient was treated with ADT before radical prostatectomy 18. Both studies have shown that pTURP can completely remove tumor lesions in the prostate, or ADT after pTURP also further inhibits the growth of residual tumors.

In our study, serum testosterone in group A recovered after discontinuing ADT treatment, but did not return to the baseline before treatment. This may be related to the fact that the testosterone level of the elderly with a longer ADT period remained castrated 19. Serum alkaline phosphatase and prostate acid phosphatase in the two groups did not change significantly before and after treatment. It showed that the overall performance of ADT treatment was no disease progression, whether intermittent or persistent.

ADT treatment can benefit patients' survival 20. A retrospectively reviewed men with localized prostate cancer who had achieved a good initial response to primary ADT and stopped it thereafter. The ADT duration and follow-up period after ADT cessation was 10 to 162 months (median, 33.5 months) and 24 to 95 months (median, 37 months), respectively. PSA recurrence was observed in 10 of 34 patients (29.4%), and the 5-year PSA progression-free rate was 66.2%. They suggested ADT can be stopped for men with localized prostate cancer, especially elderly men, after a favorable response to primary ADT 21. In our study, it was observed that after TURP, intermittent ADT was used as adjuvant treatment, with an average of 23.93 months. There was no significant difference in PSA between the two groups each year after treatment, which indicated that intermittent ADT post-pTURP could also achieve the purpose of reducing PSA by continuous ADT, and the overall treatment cost was lower than that of continuous treatment.

In our study, the 5-year cumulative survival rate of both groups was relatively high, and there was no difference between the two groups indicating that both treatments can have survival benefits.

However, anti-androgen treatment can lead to decreased bone density and increased bone fragility. Bone density in the hip, spine and distal radius was reduced by 2% to 4%, 2% to 5% and 5.3% respectively, which increases the risk of fracture by 2 to 3 times. Therefore, intermittent anti-androgen therapy can avoid the decrease of bone mineral density and benefit the patients 22. In our study, two cases of fractures were observed in patients with continuous ADT treatment in group B, which also supports this viewpoint.

In our study, the serum PSA of nine cases in group A was less than 4 ng/ml, and MRI and CT showed no local recurrence and metastasis. The 5-year PSA progression-free survival rate of group A (60%) was significant more than that of group B (26.7%). It showed a lower risk of progression to castrated resistant prostate cancer. This suggests that testosterone, which is slightly higher than castration level, may not promote the increase of PSA. This is similar to the conclusion of a long-term testosterone undecanoate replacement therapy. This study believed that Caucasians have a tendency towards increased PSA after one year of treatment compared with South Asians. But neither group had a significant risk of prostate cancer 23.

This suggests that pTURP can not only relieve urinary tract obstruction, but also completely remove localized prostate cancer tissue. Alternatively, after pTURP reduces the tumor volume, further ADT treatment is performed to eliminate the residual tumor, thereby achieving a curative effect.

Looking back at these elderly patients, we found that the efficacy exceeded our expectation. Some of them have achieved long term tumor-free survival. The patients and their families clearly expressed their confidence in our treatment plan. It is the common pursuit of doctors and patients to cure tumors, solve the problem of urination, and improve the quality of life by means of minimally invasive or combined with drugs. We have not fully understood its therapeutic mechanism. Maybe it's due to different races, genetic mutations, etc. Due to the small sample size, our results could only be used as treatment observation data for small sample size statistical calculations in a single center. A larger sample of prospective research is needed for further research.

Regarding the novelty of the research, we have two points to state.

The first point is the research object. The subject of this study is patients with localized prostate adenocarcinoma in stages T1 and T2, which belong to a relatively early stage. On the contrary, the other scholars in the literature have studied patients in advanced stages.

The second point is research objectives. This article is based on a 5-year follow-up observation that some patients have been cured through the treatment of this protocol and do not require continuous treatment or further treatment. To further clarify the possible mechanisms by which this treatment plan can enable some patients to achieve a cure outcome, rather than merely achieving low-level goals that can improve patient symptoms.

These two points are new ideas different from other studies in this article.

## Conclusions

In summary, pTURP for elderly patients with localized prostate adenocarcinoma in stages T1 and T2 with BPH combined with intermittent ADT is an effective treatment. It is able to solve dysuria as soon as possible. The overall ADT time is short. The risk of progression to castrated resistant prostate adenocarcinoma is low. Some of them have achieved tumor-free survival.

## Figures and Tables

**Figure 7:**
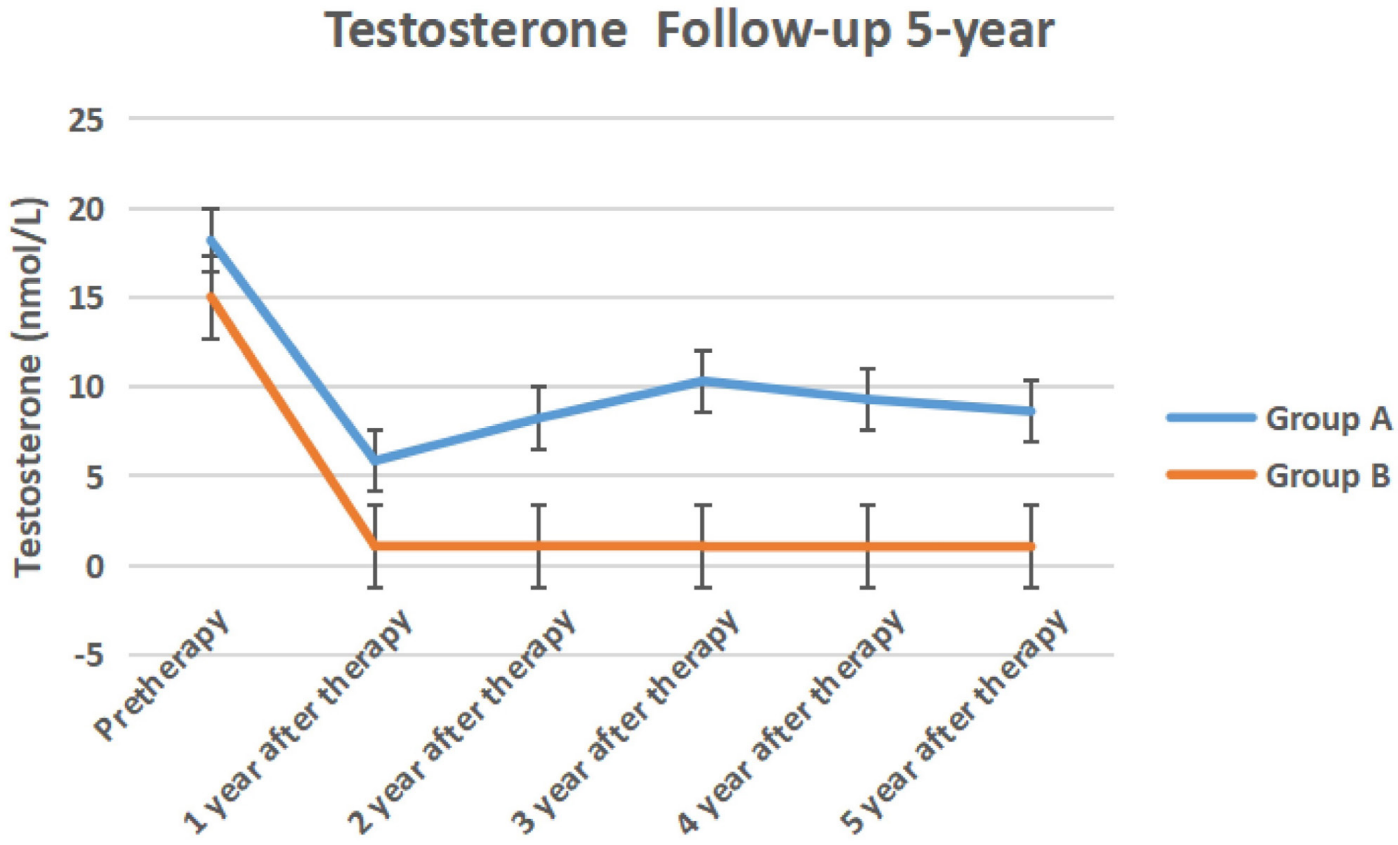
The serum testosterone of the two groups of 5-year follow-up.

**Figure 8:**
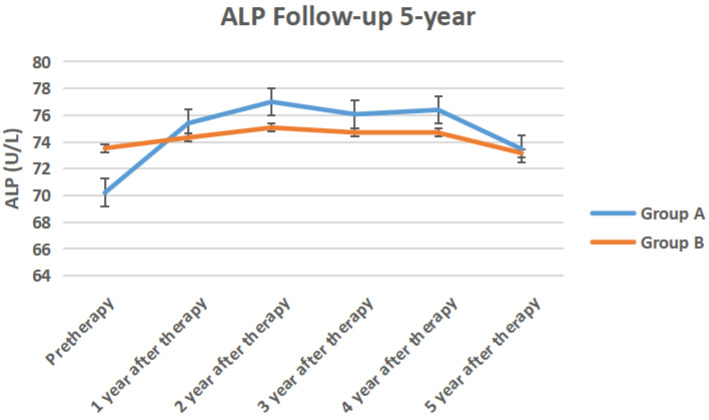
The serum ALP of the two groups of 5-year follow-up.

**Figure 11:**
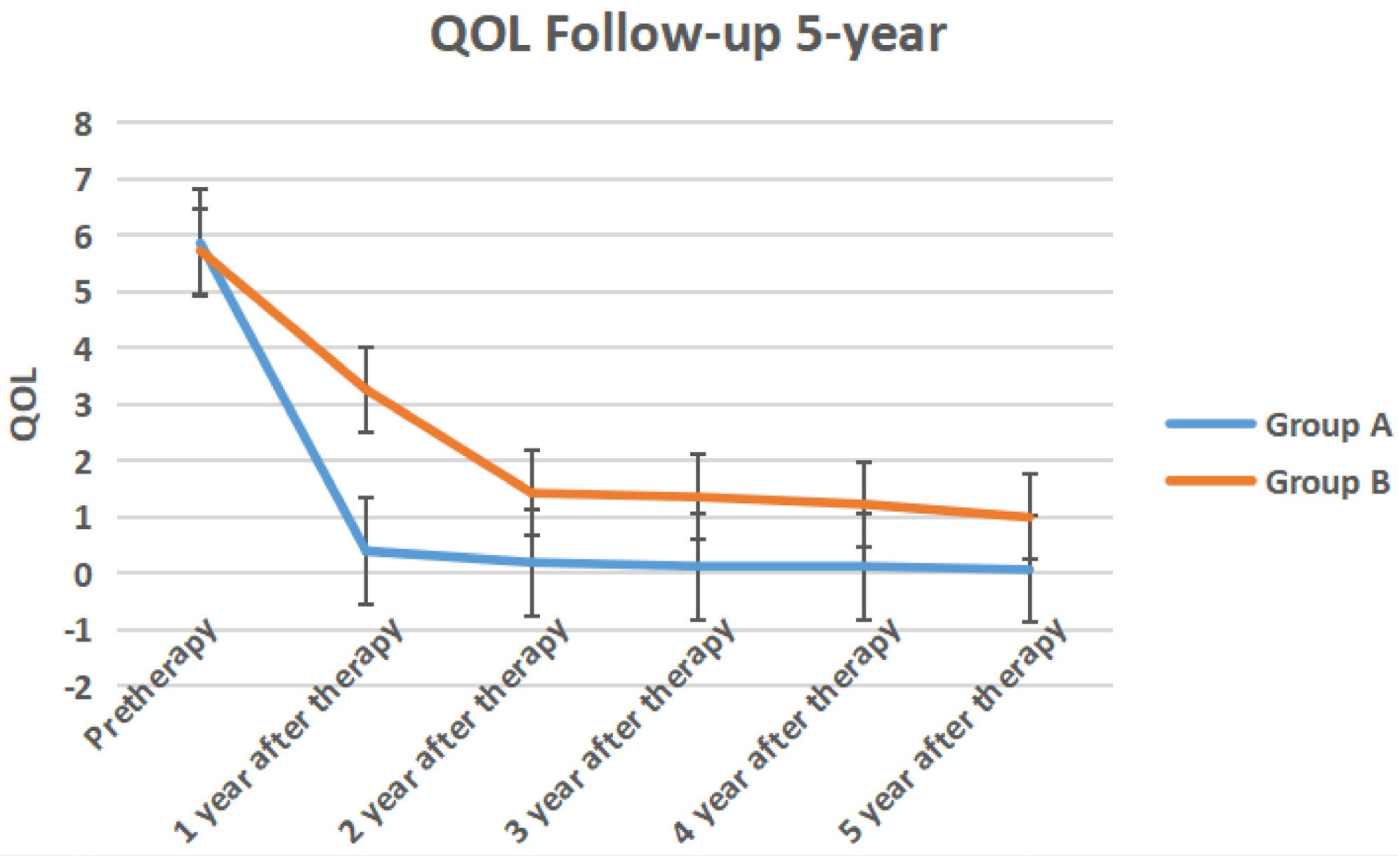
The QOL score of the two groups 5-year follow-up.

**Figure 12:**
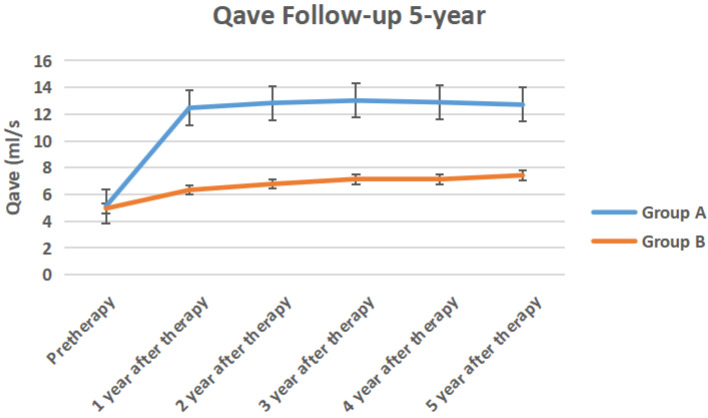
The Qmax of the two groups of 5-year follow-up.

**Figure 13:**
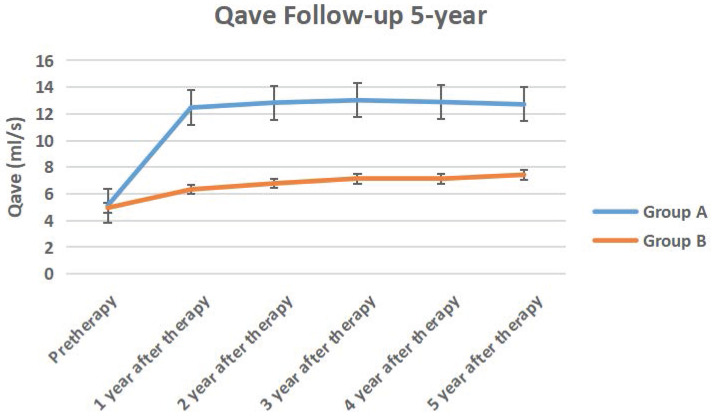
The Qave of the two groups of 5-year follow-up.

**Figure 14:**
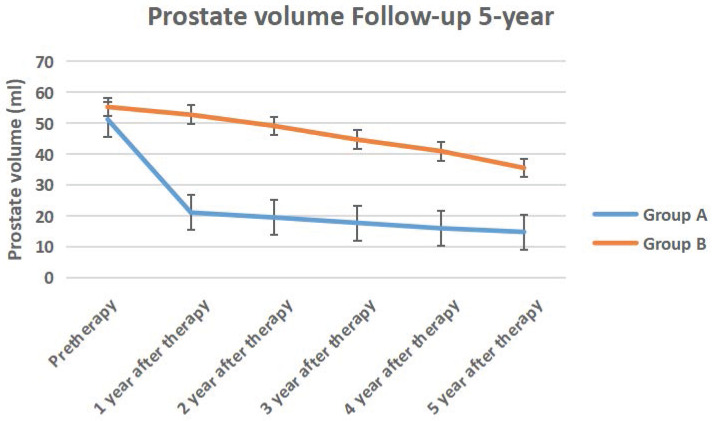
The prostate volume of the two groups of 5-year follow-up.

**Table 1:** Baseline clinical characteristics of patients before treatment

	Groupe A (n=15)	Groupe B (n=15)	*P* value
Age (y)	78.47±1.27	78.07±1.46	1.000
Gleason score (n) 3+3=6 3+4=7 4+4=8	5 4 6	4 6 5	0.811
TNM stages (n) T1N0M0 T2N0M0	1 14	2 13	1.000
Bladder stone (n)	1	0	0.483
Hypertension (n)	6	8	1.000
Diabetes (n)	2	3	1.000
Chronic bronchitis (n)	7	6	0.715
Cardiac function (n) Level I Level II Level III	4 7 4	3 6 6	0.805
Chronic renal insufficiency (n)	3	5	0.682
Sequelae of cerebrovascular (n)	1	1	1.000
Post-operative lung cancer (n)	1	0	0.483

## Abbreviations

pTURP: palliative transurethral resection of prostate
ADT: androgen deprivation therapy
BPH: benign prostatic hyperplasia
PSA: prostate specific antigen
TPSA: total prostate specific antigen
ALP: alkaline phosphatase
PAP: prostate acid phosphatase
IPSS: International Prostate Symptom Score
QOL: Quality of Life
Qmax: maximum urinary flow rate
Qave: average urinary flow rate
PVR: post void of residual urine volume
RBC: erythrocyte
WBC: leukocyte
